# Adhesive Capsulitis Following Improper Tetanus-Diphtheria (Td) Booster Administration

**DOI:** 10.7759/cureus.57113

**Published:** 2024-03-28

**Authors:** David Weinberg, Mackinzie McDaniel, Jason Pan

**Affiliations:** 1 Physical Medicine and Rehabilitation, University of Pennsylvania, Philadelphia, USA

**Keywords:** improper vaccination, booster vaccine, vaccination, diphtheria, tetanus, td, sirva, adhesive capsulitis

## Abstract

Adhesive capsulitis following vaccination is a rare complication secondary to improper intramuscular (IM) deltoid vaccine administration. It is considered a subset of the broad category known as shoulder injury related to vaccine administration (SIRVA). SIRVA typically results from improper shoulder anatomic localization prior to injection, leading to erroneous placement of the needle into the glenohumeral joint capsule or subacromial space. This can trigger a wide array of pathologies, including adhesive capsulitis.

We present the first known case of adhesive capsulitis following improper tetanus-diphtheria (Td) vaccine administration. The patient, a previously healthy middle-aged female, began experiencing significant anterior left shoulder pain the day following a Td booster vaccination. She remarked receiving the injection “higher up” in the shoulder than normal. Over the next two weeks, she began noting significant shoulder stiffness, which was followed by a progressive loss of shoulder range of motion. Her symptoms persisted for four months without definitive diagnosis or treatment. After four months of symptoms, the patient visited an outpatient sports medicine clinic where the diagnosis of adhesive capsulitis was made. Although the patient was referred for physical therapy, focusing on gentle range of motion (ROM) and stretches, followed by a planned isometric strengthening program once ROM improved, she was eventually lost to follow-up, and her recovery is unclear. Given the rarity of the diagnosis, it is unclear if adhesive capsulitis, secondary to improper IM vaccination, follows the same temporal course as “classic” adhesive capsulitis or results in a different timeframe of recovery. Further studies are needed on this subject.

## Introduction

Shoulder injury related to vaccine administration (SIRVA) is a rare complication of an intramuscular (IM) deltoid vaccine injection. It has been defined as any musculoskeletal or neurologic injury occurring within 48 hours of IM deltoid vaccine administration and persisting beyond seven days, without any symptoms prior to vaccination [1-3]. It is largely suspected to be a result of improper IM deltoid vaccine placement and/or local reaction to the injection [2-5]. Resulting pathologies, considered part of SIRVA, are broad and can include adhesive capsulitis, rotator cuff tendinitis/tear, subacromial bursitis, axillary neuropathy, septic arthritis, cellulitis, and myositis [3-5]. There is limited data regarding the incidence of SIRVA; however, one study examining COVID-19 vaccinations and SIRVA found the incidence to be ~2 cases per 10 million [4]. The incidence of adhesive capsulitis after improper vaccination, specifically, is unknown.

Cases of SIRVA have been reported following a multitude of vaccinations, primarily COVID-19. However, cases have also been reported following influenza, tetanus-diphtheria (Td), pneumococcal, human papillomavirus, and herpes simplex virus vaccinations [3,5]. There appears to be an increased likelihood of SIRVA following vaccines which require sequential booster or annual administrations [3]. Although there have been prior case reports documenting subacromial/subdeltoid bursitis following Td vaccination, there have been no documented cases, to our knowledge, resulting in adhesive capsulitis post-Td administration. According to the FDA, the most common side effects of Td vaccination are injection site pain, headaches, myalgias, injection site swelling, and erythema [6]. These adverse events are typically transient in nature, lasting an average of three days, compared to the persistent nature of SIRVA [6].

Adhesive capsulitis typically follows a three-stage pattern of symptoms - an initial “freezing” phase, followed by a “frozen” phase, and eventually resolving with a “thawing” phase. During the initial “freezing” phase, the patient experiences increased pain in the affected shoulder, which usually lasts between two and nine months [7,8]. This is followed by a progressive loss of active and passive shoulder range of motion (ROM) during the “frozen” phase, which can last between four and 12 months [7,8]. The patient finally progresses to the “thawing” phase, where there is a gradual return of active and passive ROM [7,8]. Patients typically demonstrate full recovery without treatment; however, corticosteroid injections and physical therapy can reduce symptom burden and improve ROM [7,8]. It is unclear, however, if vaccine-induced adhesive capsulitis follows this recovery pattern.

## Case presentation

A 51-year-old female with no pertinent past medical history presented to a sports medicine clinic with persistent left anterior shoulder pain for four months duration. The patient reported the pain began approximately 24 hours after receiving a tetanus-diphtheria (Td) booster vaccination. She noted she received the injection “rather high up” in her left shoulder but did not have any further specifics of the technique used. Initially, the patient’s left shoulder pain was exacerbated with overhead activities. Over the following two weeks, her shoulder pain gradually worsened and she reported stiffness in her left shoulder during active shoulder abduction and flexion. This was followed by a progressive loss of active range of motion (ROM) in all planes of motion over the following weeks. She also reported substantial left shoulder discomfort at night and experienced significant difficulty with her activities of daily living (ADLs) secondary to stiffness and diminished shoulder mobility. The patient visited urgent care soon after symptoms began, where she was advised to ice the shoulder after prolonged activities and avoid painful movements. This resulted in no improvement. After two months of symptoms, she visited her primary care provider who prescribed a short course of oral prednisone and provided her with an arm sling for comfort. The patient described no relief of her symptoms following oral prednisone and that her symptoms worsened following the use of the sling, with increasing stiffness reported with overhead shoulder movements.

Given her persistent symptoms, she presented to the sports medicine clinic four months after symptom onset. On initial examination, the active ROM of her left shoulder was approximately 90 degrees of abduction, 90 degrees of flexion, and 10 degrees of external rotation (Table 1). The patient’s active internal rotation was limited by pain and was unable to be fully assessed. Passive ROM was similar and ranged to approximately 100 degrees of abduction, 110 degrees of flexion, and 20 degrees of external rotation (Table 1). Strength and sensation to light touch in all dermatomes were intact in her left upper extremity. Other shoulder-specific examination maneuvers, including Kennedy-Hawkins, Empty Can, Speed’s, and O’Brien’s tests all resulted in worsening left shoulder pain. There were no deficits in the range of motion of her right shoulder. Shoulder XR revealed only minimal degenerative changes of the left glenohumeral joint with no other acute findings. Based on the findings, the patient was diagnosed with adhesive capsulitis and referred to physical therapy. The patient declined any pharmacologic treatment. The goals of physical therapy included slowly increasing her left shoulder ROM along with anterior and posterior glenohumeral capsular stretches, followed by isometric rotator cuff and scapular strengthening once ROM improved. The patient did not follow up again in the clinic, however, the plan was to consider intra-articular corticosteroid injection if no progress with physical therapy was made.

**Table 1:** Left shoulder mobility on examination. Active and passive range of motion of left shoulder during planes of motion. Examination performed at outpatient sports medicine clinic four months after symptom onset. ROM: range of motion

Plane of movement	Active ROM	Passive ROM
Shoulder abduction	90 degrees	100 degrees
Shoulder flexion	90 degrees	110 degrees
Shoulder external rotation	10 degrees	20 degrees
Shoulder internal rotation	Limited by pain	Limited by pain

## Discussion

Given the timing of symptom onset and subsequent clinical presentation, it is suspected that improper Td booster vaccine administration was the inciting etiology for adhesive capsulitis in this patient. The patient described receiving the injection “high up” in the shoulder and subsequently developed progressive pain and stiffness in the left shoulder. It is unknown which specific formulation of Td vaccine was utilized. To our knowledge, this is the first case of adhesive capsulitis following improper Td vaccination.

The pathophysiology of adhesive capsulitis secondary to improper IM vaccination administration is not fully understood. Previous studies have found an increased expression of inflammatory mediators (interleukins, tumor necrosis factor-alpha {TNF-α}, tumor necrosis factor-beta {TNF-β}, and vascular endothelial growth factor {VEG-F}) in the joint capsule of patients with adhesive capsulitis [9]. It is postulated that improper injection of tetanus and diphtheria toxoids into the glenohumeral joint capsule leads to a significant inflammatory response within the joint, triggering adhesive capsulitis. However, this has not been specifically studied. There is also a noted increased incidence of SIRVA in vaccines that require subsequent booster injections, namely COVID-19, influenza, HPV, and Td [3]. It may be possible that an exaggerated immune response occurs following antigen recognition in subsequent booster vaccinations, increasing the risk for adhesive capsulitis and other SIRVA [3].

In this case, it is suspected that the vaccine needle was placed into either the glenohumeral joint capsule or subacromial space, instead of the deltoid muscle belly. One potential vaccination mistake, which can increase the risk of SIRVA, is pulling a patient’s shirt over their shoulder instead of removing it/rolling up the sleeve. This can potentially distort the administrator’s ability to recognize anatomic landmarks, allowing for injection too close to lateral corner of the acromion and into the subacromial space or joint capsule, depending on the angle. Additionally, carefully located landmarks reduce the risk of other SIRVAs such as a rotator cuff tear, subacromial bursitis, or peripheral nerve injury, depending upon placement. Official CDC guidance recommends palpating the acromion and injecting 2 inches below using a 22-25 gauge, 1-1.5 inch needle for all IM deltoid injections [10]. There is no mention, however, of the best methodology to help reposition clothing in order to access the deltoid injection site (i.e., rolling up the sleeve instead of pulling shirt over, especially if the patient is wearing a long-sleeve shirt). It may be beneficial to add this information to future vaccination guidance to help reduce the risk of SIRVA.

Additionally, earlier recognition of post-vaccine adhesive capsulitis may have reduced undue suffering in the patient. The patient had symptoms for over four months before the diagnosis of adhesive capsulitis was reached. Although prior meta-analyses have concluded that the average duration of adhesive capsulitis symptoms is about one year, gradual progression in intensity of physical therapy has been associated with improved functional outcomes [11,12]. The initial goal of adhesive capsulitis rehabilitation is to focus on gentle ROM and stretching, followed by isometric and eventually isotonic strengthening [11,12]. Therefore, earlier diagnosis may have facilitated earlier referral to physical therapy, which may have improved shoulder ROM and decreased pain with ADLs. If no improvement with physical therapy occurred, then an intraarticular corticosteroid injection would have been considered. Instead, the recommendation for sling immobilization and resting the shoulder may have only worsened this patient’s symptoms. Prompt diagnosis of adhesive capsulitis ideally would have facilitated an expedited treatment plan, potentially alleviating some of the functional deficits and improving quality of life in the interim. Therefore, it is important for clinicians to consider vaccine-induced adhesive capsulitis in their differential diagnosis, especially when faced with a recently vaccinated patient who has progressively worsening mobility, stiffness, and pain in the ipsilateral shoulder of the injection site. Unfortunately, many patients lost to follow-up, including the patient in this case, so it is difficult to assess long-term functional outcomes. Moreover, given the rapid onset of symptoms post-vaccination, it is unclear if vaccine-induced adhesive capsulitis follows the classic “freezing, frozen, and thawing” phases which is the typical hallmark of adhesive capsulitis.

Ultimately, adhesive capsulitis secondary to improper vaccination, a type of SIRVA, is an uncommon and understudied diagnosis. Further research is warranted to evaluate effective treatment modalities and help inform the trajectory of healing compared to traditional-onset adhesive capsulitis.

## Conclusions

Adhesive capsulitis is an uncommon complication following improper IM deltoid vaccine administration. To our knowledge, this is the first case of adhesive capsulitis following improper Td booster administration. Our patient presented with four months of pain and diminished shoulder mobility before the diagnosis was made at an outpatient sports medicine clinic. It is imperative that vaccine administrations recognize anatomic landmarks before injection to reduce the risk of SIRVA. Moreover, it is crucial to enhance clinician awareness of SIRVA, particularly at a primary care or urgent care level, to help increase diagnostic accuracy and reduce symptom burden in patients. Long-term outcomes of adhesive capsulitis secondary to improper vaccination are unknown. Further studies are needed to better understand the pathophysiology of vaccine-induced adhesive capsulitis, the time course of symptoms, and the most effective treatment remedies.
